# Adalimumab for refractory peripheral ulcerative keratitis

**DOI:** 10.1007/s12348-012-0080-z

**Published:** 2012-05-16

**Authors:** Miguel Cordero-Coma, Raquel Salazar Méndez, Álvaro Casado Blanco, Ana López Corral, Sara Calleja-Antolín, José M. Ruiz de Morales

**Affiliations:** 1 Uveitis Unit, Hospital Universitario de León, León, Spain; 2 Department of Ophthalmology, Hospital Universitario de León, León, Spain; 3 Department of Ophthalmology, Hospital Central de Asturias, Oviedo, Spain; 4 Department of Ophthalmology, Hospital Clínico Universtario de Salamanca, Salamanca, Spain; 5 Department of Immunology, Hospital Universitario de León, León, Spain; 6 Servicio de Oftalmología, Hospital de León, c/Altos de Nava s/n, León, 24701 Spain

## Introduction

Peripheral ulcerative keratitis (PUK) is an immune-mediated disorder characterized by patchy, peripheral stromal thinning and infiltrates which tend to coalesce and extend to the limbus and often the sclera (unlike Mooren's ulcer) even in the presence of an intact overlying epithelium [1]. PUK is usually associated with systemic disorders such as rheumatoid arthritis, inflammatory bowel disease, or panarteritis nodosa among others.

Adalimumab is a human monoclonal antibody against tumor necrosis factor-alpha (TNF-α) that has demonstrated substantial efficacy when used for the treatment of several immune-mediated diseases [2, 3]. We describe two patients with refractory unilateral PUK associated with different systemic conditions who were successfully controlled with adalimumab for at least 1 year.

## Case reports

### Patient 1

A 37-year-old Caucasian male complaining of pain and redness affecting his left eye presented to our institution. He had a 7-year history of ulcerative colitis which had been treated with several systemic immunosuppressive agents including corticosteroids, azathioprine, and cyclosporine A. He also received two infusions of infliximab without response. Considering his poor response to medical therapy, a total colectomy had been performed 2 years before without further relapses of his condition.

After an unremarkable review of systems, a diagnosis of PUK was made and treatment with topical and systemic steroids (1 mg/kg/day) and subcutaneous methotrexate (initially 12.5 mg/week, increased to 15 mg/week thereafter) was initiated. Systemic steroids were slowly tapered in the following months and finally discontinued after 4 months, but the patient suffered a new relapse of his condition while on subcutaneous methotrexate (15 mg/week). Adalimumab (40 mg/2 weeks) was then initiated and methotrexate was discontinued with prompt control of his ophthalmic condition. Fifteen months after initiation of therapy with ADL, the patient remains under control.

### Patient 2

A 51-year-old male complained of pain, redness, and decrease vision affecting his left eye. He had a past medical history of rheumatoid arthritis and bilateral congenital cataracts. His arthritis had been considered as inactive for the last 2 years by his rheumatologist and remained inactive during the ophthalmologic process.

**Fig. 1:**
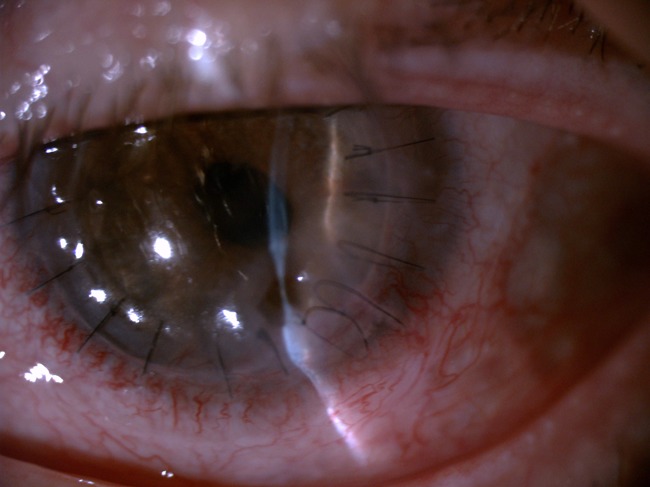
Slit lamp examination of patient 2 after the second tectonic penetrating keratoplasty. Observe the relapse of the peripheral ulcerative keratitis with corneal ulceration located at the same place of initial onset

**Fig. 2:**
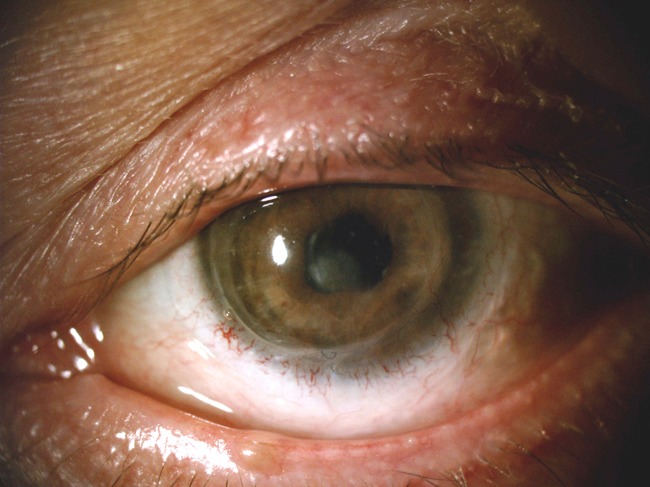
Slit lamp examination of patient 2 12 months after the initiation of therapy with adalimumab. Observe a complete resolution of the underlying inflammation and a residual corneal thinning

A thorough review of systems was unremarkable and he was diagnosed with PUK and initially treated with several systemic immunosuppressive agents including corticosteroids (1 mg/kg/day during relapses with further tapering) combined first with leflunomide (20 mg/day) and secondly with methotrexate without success. Two tectonic penetrant keratoplasties and several amniotic membrane graftings were also performed with subsequent relapses of his condition (Fig. 1). Therapy with infliximab (5 mg/kg at 0, 2, and 6 weeks) was then initiated but he developed an infusion reaction which required discontinuing therapy with infliximab and therapy with adalimumab (40 mg/2 weeks) was then initiated with subsequent control of his inflammatory condition. Twelve months after the initiation of therapy, the patient remains asymptomatic (Fig. 2).

## Discussion

We describe herein the first two reported cases of patients affected by PUK in the context of other associated immune-mediated systemic conditions who were successfully controlled with adalimumab. These two cases were particularly interesting since their systemic conditions remained under control during the inflammatory process affecting their ocular surface. In this context of unilateral PUK and systemic-associated disease under control, a local management of PUK including topical steroids and antibiotics and even surgical therapy with conjunctival resection of the inflamed area and amniotic membrane grafting would seem a good therapeutic choice. However, both cases were tremendously destructive from the disease onset requiring prompt and aggressive therapy with systemic immunosuppressors. In addition, both patients were refractory to traditional immunosuppressive therapy for their respective associated conditions.

TNF-α blockers and among them adalimumab have demonstrated to be a safe and effective therapy for induction of clinical remission in patients with active ulcerative colitis and rheumatoid arthritis (the two systemic-associated diseases in these two presented cases) failing treatment with corticosteroids and/or immunosuppressants [2, 3]. TNF-α has been demonstrated to be one of the main pro-inflammatory molecules causing and/or perpetuating the PUK process [4]. In this sense, we have reported promising results when using adalimumab for Mooren's ulcer [5]. TNF-α blockers in general may be a good therapeutic option for these patients based not only in their anti-inflammatory effects, but they may also act as "remodeling" agents as observed in other diseases, such as rheumatoid arthritis, in which these agents contribute to modulate systemic and local bone resorption and reduce cartilage degradation [6].

Infliximab is a chimeric monoclonal antibody against TNF-α that has been successfully used for the treatment of PUK [7, 8]. However, patient 2 had an infusion reaction which required discontinuing medication. Patient 1 was an actively working young man with restrictions to get time off from work to get his infusions and therefore we chose adalimumab (instead of infliximab) because it is administered subcutaneously.

Although other anti-TNF-α agents, such as infliximab, have been successfully used for the treatment of PUK associated not only with their FDA-approved indications but also with other systemic immune-mediated conditions, it is imperative to take into account that not all patients respond to their first anti-TNF-α agent. Although these are preliminary data, it is clearly useful to have a range of effective treatment modalities available to treat patients with clinically relevant PUK. The issue of how long treatment should be continued in these patients should also be raised, although our limited experience on this therapeutic approach leaves this important question open to conjecture.

## Conclusion

These preliminary data suggest that adalimumab may be an attractive option for the treatment of refractory cases of PUK, and more definitive studies on this matter are warranted.
